# Medical Respite Care and Changes in Acute Care Use Among People Experiencing Homelessness: A Matched‐Cohort Evaluation in Edmonton, Canada

**DOI:** 10.1111/jep.70647

**Published:** 2026-11-03

**Authors:** M. H. Pulok, J. Van Nest, M. Ghosh, F. Mosaico, J. Nath, T. Smith, M. Roy, V. Saini

**Affiliations:** ^1^ Health, Intelligence, Evidence & Improvement, Primary Care Alberta Calgary Alberta Canada; ^2^ Department of Community Health Sciences University of Calgary Calgary Alberta Canada; ^3^ Edmonton Public Health, Primary Care Alberta Edmonton Alberta Canada; ^4^ Department of Medicine Faculty of Medicine & Dentistry, University of Alberta Edmonton Alberta Canada; ^5^ Radius Community Health & Healing Edmonton Alberta Canada

**Keywords:** Alberta, difference‐in‐differences, emergency department, inpatient admissions, length of stay

## Abstract

**Rationale:**

Respite to Recovery (R2R), operated by Radius Community Health & Healing, is a Medical Respite Care Programme (MRCP) in Edmonton providing short‐term residential care to individuals experiencing homelessness who are medically stable but cannot safely recuperate in a shelter or on the street. Evidence from Canadian settings remains limited.

**Objectives:**

This study evaluated the changes in acute care utilisation before and after participation in R2R (hereafter MRCP), relative to matched comparison individuals experiencing homelessness in Edmonton.

**Methods:**

We conducted a retrospective matched cohort study comparing MRCP participants with matched controls. Descriptive and difference‐in‐differences (DiD) analyses focused on emergency department/urgent care centre (ED/UCC) visits, inpatient admissions, and length of stay for ED/UCC visits and hospitalisations.

**Results:**

The cohort included 143 MRCP participants and 546 matched controls. MRCP participants had substantially higher pre‐index acute‐care utilisation despite a close balance on age and sex. DiD analyses showed larger reductions in the MRCP group for ED/UCC visits (DiD −1.22; 95% CI −2.03 to −0.42), inpatient admissions (−0.39; 95% CI −0.64 to −0.15), and inpatient days (−4.83; 95% CI −9.27 to −0.39). The ED/UCC length‐of‐stay and the person‐time‐adjusted estimates for ED/UCC and inpatient admission rates were not statistically significant.

**Conclusions:**

MRCP participation was associated with larger reductions in several acute care and burden measures, although person‐time‐adjusted between‐group differences in ED/UCC visit and inpatient admission rates were not statistically significant. Substantial pre‐index utilisation differences and divergent pre‐index trends limit causal interpretation; the findings should therefore be considered exploratory. Continued evaluation of MRCP with refined methodologies and extended follow‐up is essential to confirm these findings and guide policy decisions.

## 1 Introduction

Homelessness presents multifaceted challenges, including health disparities stemming from chronic illnesses, mental health disorders, and substance use. It is a critical public health issue, exerting substantial pressure on healthcare systems worldwide, especially in high‐income countries like Canada [1, 2, 3]. Individuals experiencing homelessness (IEH) often experience elevated rates of chronic illness, mental health conditions, and substance use disorders, alongside inadequate access to stable housing and primary care services [2, 4, 5]. Therefore, they disproportionately rely on acute care settings due to limited access, and at times, engagement with primary care and preventative health care services [2, 6, 7].

Emergency departments (ED) often become the default point of care among IEH. Estimates suggest that IEH access ED services between 1.63 and 18.75 times more frequently than individuals with stable housing [8, 9]. As a result, rather than accessing preventative care or receiving appropriate outpatient treatment, IEH frequently turn to EDs during health crises, often when their conditions have already worsened. Frequent utilisation of EDs by IEH contributes to recurrent hospital admissions and extended stays in both emergency and acute care settings [1, 10, 11, 12]. For example, about 70% of the hospitalisations among IEH result in re‐admission or ED visits within 30 days, with 75% of re‐admissions occurring within the first 2 weeks of discharge [1]. Further, the length of stay (LOS) in ED and hospital tends to be longer due to the complexity of medical and social needs, including the lack of an appropriate discharge destination [7, 13, 14].

The cyclical nature of homelessness and acute care utilisation not only strains the healthcare system but also fails to address the underlying factors contributing to poor health outcomes among IEH. Additionally, healthcare providers may face challenges in safely discharging IEH due to unstable housing situations and inadequate community support systems [15]. Consequently, hospitals experience increased patient load and resource demand, leading to overcrowding, strained healthcare personnel, and rising operational costs [16, 17]. Addressing this cycle requires a shift toward more integrated care models that emphasise continuity of care, preventive services, and comprehensive social support. Targeted interventions prioritising both the immediate medical needs and the broader social determinants of health affecting IEH are needed to extend beyond traditional healthcare settings.

Initiatives such as medical respite care programmes (MRCP) have emerged as promising solutions, offering transitional care services that bridge the gap between acute care settings and long‐term health management. MRCPs have emerged as providing short‐term residential care for IEH who are too ill or frail to recover on the streets but not ill enough to remain in a hospital [1, 18]. Typically, they are small (fewer than 20 beds) and run by non‐profit organisations, with average stay lasting two to 4 weeks [19]. These programmes provide varying levels of medical care, which may include part‐time or full‐time on‐site physicians, visiting nurses, volunteer providers, or referrals to the local clinics. In general, MRCPs offer a stable, supportive setting that facilitates recovery and incorporates services that address the complex medical and social needs of participants [20].

Previous studies from the United States of America and other countries have suggested that MRCPs may alter acute health care utilisation patterns among IEH [15]. Participants in MRCPs have lower hospital re‐admissions within 90 days compared to those who were discharged to the street or shelters [4], and other evaluations have also reported reductions in inpatient days and potential cost savings [11, 21, 22, 23, 24]. For example, a randomised controlled trial (RCT) in Denmark found significantly reduced health and social costs for patients enrolled in a 2‐week MRCP [1]. However, programme models and health‐system contexts vary, and evidence from Canadian settings remains limited.

In Edmonton, Alberta, Canada, Respite to Recovery (R2R), an MRCP was launched in October 2023 to serve IEH burdened by chronic health conditions and frequent acute care visits. R2R is operated by Radius Community Health & Healing (Radius) with funding from Alberta Health Services (AHS). In 2025, the budget was transferred to Primary Care Alberta (PCA), and PCA continues to fund the programme. The programme is based on Radius' (formerly Boyle McCauley Health Centre) 47 years of experience in operating the Community Nursing Station (CNS) at the Herb Jamieson shelter from 1972 to 2019. Radius Community Health and Healing is a non‐profit, community‐owned and operated community health centre that offers accessible, comprehensive, culturally sensitive primary care and social support to individuals who face multiple barriers to accessing health services.

R2R is the first, and currently, the only Medical Respite Unit (MRU) in Alberta and was initiated as a pilot site to support the development of a provincial MRCP. The R2R programme provides a space for IEH who are medically stable yet have healthcare needs that are too complex for independent management in a shelter setting or on the streets, but not serious enough to require emergency care or hospitalisation. While staying in the MRU, participants were offered the opportunity to engage in primary care offered through Radius. R2R staff supported participants through the attachment process, ensuring that previously unattached patients were discharged from the MRU with a primary care provider in the community. Additionally, R2R staff operated a walk‐in clinic out of the MRU nursing station, which provided participants with an accessible pathway to seek care and support post‐discharge.

The MRCP envisions establishing MRUs throughout Alberta, adapted to the local context; however, further expansion is contingent on the evaluation of the Edmonton programme. Such an assessment could inform future healthcare strategies and resource allocation to better serve this vulnerable group. Given the limited Canadian evidence, the objective of this study was to examine changes in acute‐care utilisation associated with the implementation of R2R (hereafter mentioned as MRCP) among IEH in Edmonton.

## 2 Materials and Methods

### 2.1 Programme Setting and Intervention

During the study period, R2R operated on weekdays with 27 beds (20 low‐mobility beds and 7 top bunks). The programme provided nurse‐led support, with access to Radius primary care physicians or nurse practitioners as needed. During patients' interactions in acute care and community settings, eligible community members were offered the opportunity to learn more about R2R and asked if they would like a referral to the MRU. If agreeable, the referring agency would contact R2R and begin the referral process to determine eligibility criteria and bed availability. If accepted, a participant was enrolled in the MRCP upon admission into the MRU. For example, in the acute care setting, the referral process would typically be facilitated through the discharge planning process. Upon referral acceptance by R2R staff, the referring agency would facilitate discharge from the hospital and transportation to the MRU. Upon arrival at the MRU, R2R staff would complete the intake process and enrol participants into the MRCP. A small proportion of participants were enrolled through presentation to the onsite walk‐in clinic.

### 2.2 Data Sources

Data on acute care utilisation were extracted from the National Ambulatory Care Reporting System (NACRS) and Discharge Abstract Database (DAD) for the eligible individuals. MRCP participation data were provided through the programme reporting process and linked to administrative health records using unique identifiers within the secure data environment. Radius Community Health & Healing submitted monthly MRCP service‐activity reports to the Primary Care & Chronic Disease Management (PCCDM) department of Edmonton Zone Community Health Services. PCCDM staff from the Vulnerable Populations Support Team verified MRCP patient records in Connect Care and linked them to the relevant acute‐care records.

### 2.3 Study Population, Period and Inclusion Criteria

At the programme level, individuals requiring basic medical care beyond self‐management but not requiring admission to or continued stay in the hospital were eligible to enrol on the MRCP. For this evaluation, homelessness was identified from DAD and NACRS using the International Statistical Classification of Diseases and Related Health Problems, Tenth Revision, Canada (ICD‐10‐CA) code Z59.0, indicating homelessness documented at the time of a healthcare encounter [25]. A qualifying Z59.0 record was required for inclusion in both the MRCP and matched control analytic cohorts. The study included adults aged 18 years or older and had at least one acute care encounter in the Edmonton zone during the study period, from 20th April 2023 to 30th June 2024. Only individuals meeting the Z59.0‐based homelessness criteria during the study period were included. Individuals with an invalid healthcare number, missing sex information, and date of birth were excluded (< 1.0%).

### 2.4 Study Design

The study employed a matched cohort study design [26]. This design entails a pre‐post comparison between IEH who accessed the MRCP (intervention/exposed group) and the matched IEH who did not access the programme (control group) during the study period. The first MRCP enrolment date served as the participant‐specific index date, and controls within each matched set were assigned the same index date. The study used a maximum 180‐day window on each side of the index date, bounded by the study start (20 April 2023) and end (30 June 2024). Utilisation on the index date was classified as pre‐index because within‐day sequencing of acute‐care use and MRCP enrolment was unavailable; the post‐index period began the following day.

### 2.5 Outcome Variables

The primary outcomes of this study were the number of ED/UCC visits, inpatient admissions, total inpatient days, i.e. length of stay (LOS in days), and mean ED/UCC length of stay (hours) among periods with at least one ED/UCC encounter. Additionally, ED/UCC visits and inpatient admissions rates were calculated per 10,000 person‐days.

### 2.6 Exposure, Covariates and Matching

The primary exposure variable was enrolment in the MRCP. As exposure to the MRCP among participants in the intervention group happened at different times during the study period, the date of enrolment in the MRCP was determined as the index date for the exposed group participants. Potential controls were matched on age (± 2 years), exact sex (male, female), and health care institutions (ED/UCC centres, hospitals) where they received services. Each selected control was then assigned the corresponding MRCP participant's index date. When more than four eligible controls were available, up to four were randomly selected using the original matching code. To preserve equal control mass across variable‐ratio matched sets, MRCP participants received weight 1 and each control within a set received weight 1 divided by the number of controls in that set. The matched controls were IEH who did not enrol in MRCP during the study period, and they were not specifically defined as individuals who declined MRCP. There was no way of knowing if controls were offered and denied admission to the MRCP.

### 2.7 Statistical Analysis

Descriptive statistics were used to summarise baseline characteristics and acute care use. Baseline balance was assessed using weighted standardised mean differences (SMDs), with values closer to zero indicating better balance. Pre‐index utilisation counts and inpatient days were standardised to a 180‐day period to account for differences in observable person‐time. Explicit person‐period records were constructed for every matched‐set membership so that periods with no ED/UCC visit or inpatient admission were retained as true zeros. Mean LOS (hours) at ED/UCC was treated as a conditional outcome of the ED/UCC visit and remained missing when no ED/UCC encounter occurred. The post‐index period included both time during MRU residence and, where applicable, time after MRU discharge; these exposure states were not analysed separately.

For each outcome, regression‐based difference‐in‐difference (DiD) models included MRCP status, post‐index period, and their interaction. Weighted linear models were used for observed‐period ED/UCC visit counts, conditional mean ED/UCC LOS, inpatient admissions, and total inpatient days. Primary frequency analyses used weighted Poisson regression for ED/UCC visits and inpatient admissions with person‐days as the exposure. Standard errors were clustered by individuals to account for repeated pre/post observations and reuse of some controls across matched sets. Marginal DiD estimates were reported on the scale of rates per 10,000 person‐days with 95% confidence intervals. Sensitivity analyses included unweighted Poisson models, minimum‐observation restrictions of 30, 60, and 90 days on both sides of the index date, and negative‐binomial models. Six 30‐day pre‐index bins were used to descriptively assess pre‐index utilisation trajectories. Statistical significance was assessed at *p* < 0.05 level. All statistical analyses were carried out using Stata/SE 15.1 [27].

## 3 Results

### 3.1 Baseline Characteristics

The matched cohort included 143 MRCP participants and 546 matched control individuals (Table 1). Age and sex were closely balanced after weighting (absolute SMD approximately 0.00). The mean age of study participants was about 47 years, with a predominance of males in both groups. In contrast, substantial pre‐index utilisation differences remained: absolute SMDs were 0.53 for ED/UCC visits, 0.21 for mean ED/UCC LOS, 0.94 for inpatient admissions, and 0.53 for inpatient days (Table 1).

**Table 1:** Baseline characteristics of the matched cohort.

	MRCP group (*n* = 143)	Matched control group (*n* = 546)	Absolute SMD
Demographic characteristic			
Age in years: weighted mean	46.76	46.78	0.00
Sex			0.00
Male: weighted %	77.62	77.62	
Female: weighted %	22.38	22.38	
Pre‐index acute‐care utilisation			
ED/UCC visits per 180 days: weighted mean	3.72	0.78	0.53
Mean ED/UCC LOS (hours): weighted mean	15.38	12.81	0.21
Inpatient admissions per 180 days: weighted mean	1.11	0.15	0.94
Inpatient days per 180 days: weighted mean	14.41	3.25	0.53

*Note:* Values are weighted to account for the variable number of controls within matched sets. Mean ED/UCC LOS is conditional on having at least one ED/UCC encounter during the pre‐index period. The 546 unique control individuals contributed 569 control‐set memberships because some controls were reused across matched sets.

Abbreviations: ED, emergency department; LOS, length of stay; MRCP, Medical Respite Care Programme; SMD, standardised mean difference; UCC, urgent care centre.

### 3.2 Change in the Intervention (MRCP) Group

Among MRCP participants, all six descriptive utilisation measures decreased after enrolment (Table 2). Mean ED/UCC visits decreased from 3.72 to 2.20, mean ED/UCC LOS from 15.38 to 12.66 h, inpatient admissions from 1.11 to 0.65, and inpatient days from 14.39 to 7.26. ED/UCC and inpatient admission incidence rates also declined, by 30.53 and 9.76 events per 10,000 person‐days, respectively. These within‐group changes are descriptive and should not be interpreted as programme effects in isolation.

**Table 2:** Acute care use before and after MRCP enrolment among MRCP participants.

Outcome	Pre‐index	Post‐index	Change*
ED/UCC visits: mean (SD)	3.72 (7.44)	2.20 (4.74)	−1.52
Mean ED/UCC LOS (hours): mean (SD)*	15.38 (9.13)	12.66 (6.64)	−2.72
Inpatient admissions: mean (SD)	1.11 (1.37)	0.65 (1.13)	−0.46
Inpatient days: mean (SD)	14.39 (21.99)	7.26 (16.45)	−7.13
ED/UCC incidence rate (per 10,000 person‐days)	206.99	176.46	−30.53
Inpatient incidence rate (per 10,000 person‐days)	61.86	52.10	−9.76

* Change = post‐index minus pre‐index. Descriptive estimates only; inferential results are reported in Table 4.

### 3.3 Change in the Control Group

Matched controls also showed lower acute‐care utilisation after the assigned index date (Table 3). Mean ED/UCC visits decreased from 0.78 to 0.48, mean ED/UCC LOS from 12.81 to 11.41 h, inpatient admissions from 0.15 to 0.08, and inpatient days from 3.23 to 0.92. ED/UCC and inpatient admission incidence rates declined by 4.61 and 1.94 events per 10,000 person‐days, respectively. The presence of declines in both groups reinforces the need for between‐group DiD comparisons.

**Table 3:** Acute care use before and after among controls following the matched index date.

Outcome	Pre‐index	Post‐index	Change*
ED/UCC visits: mean (SD)	0.78 (2.53)	0.48 (1.86)	−0.29
Mean ED/UCC LOS (hours): mean (SD)	12.81 (14.79)	11.41 (10.24)	−1.40
Inpatient admissions: mean (SD)	0.15 (0.45)	0.08 (0.31)	−0.07
Inpatient days: mean (SD)	3.23 (20.07)	0.92 (6.34)	−2.31
ED/UCC incidence rate (per 10,000 person‐days)	43.12	38.51	−4.61
Inpatient incidence rate (per 10,000 person‐days)	8.11	6.16	−1.94

* Change = post‐index minus pre‐index. Descriptive estimates only; inferential results are reported in.

**Table 4:** Difference‐in‐differences estimates of changes in acute‐care utilisation.

Outcome	MRCP group change	Control group change	DiD estimate (95% CI)	*p*‐value
No. of ED/UCC visits: mean	−1.52	−0.29	−1.22 (−2.03 to −0.42)	0.003
Length of stay in ED/UCC (hours): mean	−2.72	−1.40	−1.31 (−5.30 to 2.67)	0.518
No. of inpatient admissions: mean	−0.46	−0.07	−0.39 (−0.64 to −0.15)	0.002
Inpatient days: mean	−7.13	−2.31	−4.83 (−9.27 to −0.39)	0.033
ED/UCC incidence rate (per 10,000 person‐days)	−30.53	−4.61	−25.92 (−67.21 to 15.37)	0.219
Inpatient incidence rate (per 10,000 person‐days)	−9.76	−1.94	−7.82 (−23.23 to 7.59)	0.320

* DiD estimate = MRCP change ‐ control change. The first four rows are weighted linear DiD contrasts; rate rows are marginal DiD contrasts from weighted Poisson models using person‐days as the exposure. Standard errors are clustered by individual.

### 3.4 Difference in Change Between Intervention (MRCP) and Control Group (Difference‐In‐Differences)

Figure 1 provides a descriptive assessment of utilisation trajectories during six consecutive 30‐day periods before the index date. The patterns do not support parallel pre‐index trends: ED/UCC visit and inpatient admission rates were substantially higher in the MRCP group and increased sharply in the period immediately preceding enrolment, whereas control rates were comparatively stable. This is consistent with the selection of individuals with higher acute‐care needs into MRCP and raises the possibility of regression to the mean. Accordingly, the DiD estimates are interpreted as comparisons of observed change rather than definitive causal effects [28, 29].

**Figure 1:**
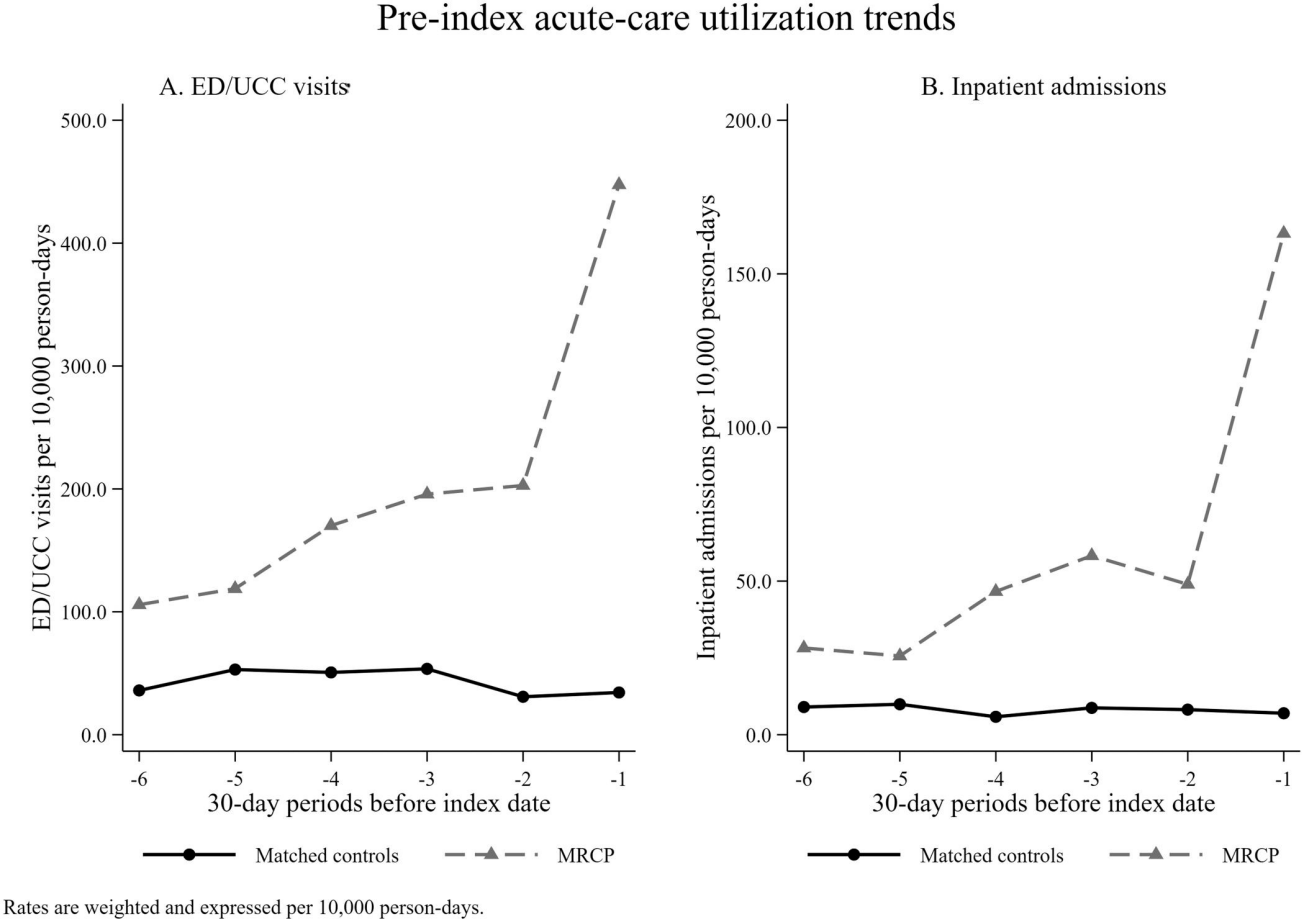
Pre‐index acute‐care utilisation trends among MRCP participants and matched controls.

In weighted linear DiD analyses, reductions in observed counts or burden were greater in the MRCP group for ED/UCC visits (DiD −1.22; 95% CI −2.03 to −0.42; *p* = 0.003), inpatient admissions (−0.39; 95% CI −0.64 to −0.15; *p* = 0.002), and inpatient days (−4.83; 95% CI −9.27 to −0.39; *p* = 0.033) (Table 4). The between‐group difference in mean LOS of ED/UCC was not statistically significant (DiD −1.31 h; 95% CI −5.30 to 2.67; *p* = 0.518). In person‐time‐adjusted Poisson analyses, the marginal DiD was −25.92 ED/UCC visits per 10,000 person‐days (95% CI −67.21 to 15.37; *p* = 0.219) and −7.82 inpatient admissions per 10,000 person‐days (95% CI −23.23 to 7.59; *p* = 0.320); neither rate‐based estimate was statistically significant. Sensitivity analyses were consistent with these rate‐based findings (Supplementary Table S1). Table 4 reports additive marginal rate differences, whereas Table S1 reports multiplicative interaction estimates (ratio of rate ratios) from the corresponding count models. Given the substantial differences in baseline utilisation rates, additive marginal rate differences and multiplicative interaction estimates need not indicate the same relative direction; neither person‐time‐adjusted estimate provided statistically significant evidence of a differential post‐index change.

## 4 Discussion

This study examined changes in acute healthcare utilisation associated with enrolment into a pilot MRCP among the IEH in Edmonton. By comparing MRCP participants with the matched controls before and after participant‐specific index dates, the study identified changes in ED/UCC and inpatient utilisation. The findings are mixed: observed counts and inpatient‐day burden declined more in the MRCP group than among controls, whereas person‐time‐adjusted incidence‐rate differences for ED/UCC visits and inpatient admissions were not statistically significant.

Matching achieved close balance on age and sex, but important differences remained in pre‐index acute‐care utilisation. The mean age of participants (about 47 years) and the predominance of males were broadly consistent with populations of IEH accessing acute care in previous studies [1, 7, 24]. However, MRCP participants had substantially higher ED/UCC use, inpatient admissions, and inpatient days before enrolment, with the largest imbalance for inpatient admissions. Higher baseline utilisation among respite populations has also been described elsewhere [4, 24]. This pattern is consistent with the possible selection of individuals with greater medical and social care needs into the programme, aligning with the population targeted by medical respite care [15, 23]. The baseline differences should be carefully considered when interpreting the findings of this study.

Among MRCP participants, ED/UCC visits, ED/UCC LOS, inpatient admissions, inpatient days, and the corresponding incidence rates all declined descriptively after enrolment. DiD analyses suggested larger reductions in ED/UCC visits, inpatient admissions, and inpatient days than among controls. However, the person‐time‐adjusted rate analyses did not show statistically significant differences in ED/UCC or inpatient admission incidence. These mixed findings are broadly compatible with prior studies reporting lower inpatient utilisation after medical respite care [5, 21, 22, 24], but they do not establish the same effect in the present evaluation.

Descriptive results showed that ED/UCC visit counts declined among MRCP participants, and DiD analysis indicated a larger reduction than among matched controls; however, the person‐time‐adjusted analysis did not identify a statistically significant between‐group difference in ED/UCC visit rates. Similarly, although the mean ED/UCC length of stay declined after MRCP enrolment, the between‐group DiD estimate was not statistically significant. These findings suggest a potential trend toward reduced acute care use post‐MRCP exposure, which is consistent with literature indicating that medical respite care can decrease acute care utilisation and associated healthcare costs [5, 21, 22, 24].

One plausible mechanism for shorter acute‐care stays is that MRCP provides a stable recuperative setting and a safer discharge destination once acute care is no longer required. Nurse‐led support and access to primary care clinicians may also influence the timing and threshold for return to ED/UCC. These mechanisms are consistent with the rationale for medical respite programmes [15, 16], but they were not measured directly in this study. The marked increase in MRCP utilisation immediately before enrolment further cautions against attributing the subsequent decline solely to the programme.

The key strength of this study is the use of linked administrative data and a matched cohort design to evaluate a real‐world programme serving a population that is difficult to study prospectively. Matching improved comparability on the measured matching variables, but still did not eliminate important baseline utilisation differences or unmeasured confounding. Leveraging AHS's population‐level administrative data also ensured access to high‐quality, objective, and comprehensive records for all participants.

The study findings are subject to several limitations. First, a substantial pre‐index utilisation imbalance and the sharp increase in MRCP utilisation near enrolment suggest programme selection and regression to the mean. Second, unmeasured confounders, including mental health status, substance use, disease severity, social support, and housing trajectory, may have influenced the observed associations. Third, treatment timing varied across participants, producing different amounts of observable follow‐up; person‐time was incorporated into the rate models, but shorter windows limit assessment of sustained change. Fourth, the pre‐index trends were not parallel, weakening the identifying assumptions required for a causal DiD interpretation.

The study was conducted in Edmonton, and the findings may not be generalised to other contexts with different IEH population dynamics, acute health care services, or programme structures. From a quality improvement lens, the MRCP was only open on weekdays and had limited bed capacity. This limited the intake and transfer of individuals from acute care, which could have impacted the results. The evaluation did not distinguish utilisation occurring during MRU residence from utilisation after discharge, and ED/UCC LOS was conditional on having an encounter. Clinical outcomes, housing outcomes, duration of MRU residence, referral pathways, and post‐discharge service engagement were not measured. Future evaluations should incorporate these dimensions and extend follow‐up to assess whether observed utilisation changes are sustained.

## 5 Conclusion

This matched‐cohort evaluation found that MRCP participation was associated with larger reductions in several observed acute‐care count and burden measures, including ED/UCC visits, inpatient admissions, and inpatient days. However, person‐time‐adjusted between‐group changes in ED/UCC and inpatient admission rates were not statistically significant, and pre‐index utilisation differed substantially between groups and diverged near enrolment. The findings, therefore, suggest potentially important changes in utilisation but do not establish a causal effect of MRCP. Continued evaluation of the MRCP with longer follow‐up, richer clinical and social information, and stronger methods to address treatment selection is warranted to confirm these findings and guide future policy decisions.

## Author Contributions

M. H. Pulok, J. Van Nest, M. Ghosh and V. Saini contributed to the conceptualisation and study design. Material preparation, data extraction and statistical analysis were performed by M. H. Pulok and M. Roy assisted in data analysis and method development. The first draft of the manuscript was written by M. H. Pulok and all authors commented on the previous versions of the manuscript. T. Smith, M. Ghosh, J. Nath, F. Mosaico, J. Van Nest, and V. Saini critically reviewed and edited the final version of the manuscript. All authors read and approved the final version of the manuscript.

## Funding

The authors have nothing to report.

## Ethics Statement

This study was performed in line with the principles of the Declaration of Helsinki. Ethics approval was obtained from the Conjoint Health Research Ethics Board at the University of Calgary (Ethics ID: REB25‐0229). A waiver of consent was provided to access administrative health records from Alberta Health Services.

## Consent

The authors have nothing to report.

## Conflicts of Interest

The authors declare no conflicts of interest.

1

This study used Stata/SE 15.1 for all the statistical analyses. Codes are available from the authors at reasonable request.

## Supporting information


Supporting File

## Data Availability

Data used in this study is held securely at Alberta Health Services. Although data‐sharing agreements prohibit Alberta Health Services from making the dataset publicly available, access may be granted to those who meet pre‐specified criteria for confidential access. Please contact [research.administration@ahs.ca] for more information. The views and opinions expressed there are solely of the authors and do not necessarily represent official positions of Alberta Health Services, Primary Care Alberta, Radius Health and the University of Calgary.
