# Supplementary material for: Medical Respite Care and Changes in Acute Care Use Among People Experiencing Homelessness: A Matched‐Cohort Evaluation in Edmonton, Canada
Source: J Eval Clin Pract. 2026 Nov 3;32(8):e70647. doi: 10.1111/jep.70647 (PMC13730316; doi:10.1111/jep.70647)
Supplement: Supplementary file 1 [file JEP-32-0-s001.docx]

**Supplementary materials**

**Table S1. Sensitivity analyses for incidence-rate models**

| **Model** | **Ratio of rate ratios** | **95% CI** | **P value** | **N** |
| --- | --- | --- | --- | --- |
| Weighted Poisson: ED/UCC | 0.95 | 0.65 to 1.40 | 0.812 | 1,424 |
| Weighted Poisson: inpatient | 1.11 | 0.67 to 1.83 | 0.688 | 1,424 |
| Unweighted Poisson: ED/UCC | 0.96 | 0.65 to 1.40 | 0.821 | 1,424 |
| Unweighted Poisson: inpatient | 1.11 | 0.68 to 1.84 | 0.669 | 1,424 |
| ED/UCC Poisson; >=30 days both periods | 0.92 | 0.64 to 1.33 | 0.658 | 1,304 |
| Inpatient Poisson; >=30 days both periods | 1.08 | 0.65 to 1.80 | 0.758 | 1,304 |
| ED/UCC Poisson; >=60 days both periods | 0.90 | 0.63 to 1.29 | 0.572 | 1,164 |
| Inpatient Poisson; >=60 days both periods | 1.07 | 0.64 to 1.80 | 0.796 | 1,164 |
| ED/UCC Poisson; >=90 days both periods | 0.78 | 0.56 to 1.08 | 0.136 | 986 |
| Inpatient Poisson; >=90 days both periods | 0.95 | 0.54 to 1.68 | 0.860 | 986 |
| Negative binomial: ED/UCC | 1.00 | 0.69 to 1.47 | 0.980 | 1,424 |
| Negative binomial: inpatient | 1.09 | 0.67 to 1.79 | 0.727 | 1,424 |

Note: A ratio of rate ratios below 1 indicates a relatively larger post-index rate reduction in *the MRCP group*. N is the number of person-period observations, not the number of unique individuals. Standard errors for regression models were clustered by individuals.
